# Phylogenetic and phylogeographic evidence for the colonization success of the diplochorous *Astydamia latifolia* across the Canary Islands

**DOI:** 10.1002/ece3.11624

**Published:** 2024-07-04

**Authors:** Alberto J. Coello, Pablo Vargas, Aitor Alameda‐Martín, Emilio Cano, Yurena Arjona, Mario Fernández‐Mazuecos

**Affiliations:** ^1^ Departamento de Biología (Botánica) Universidad Autónoma de Madrid Madrid Spain; ^2^ Real Jardín Botánico (RJB‐CSIC) Madrid Spain; ^3^ Department of Botany National Museum of Natural History, Smithsonian Institution Washington DC USA; ^4^ Departamento de Agronomía Universidad de Almería Almería Spain; ^5^ Department of Botany, Ecology and Plant Physiology University of La Laguna San Cristóbal de La Laguna Spain; ^6^ Centro de Investigación en Biodiversidad y Cambio Global (CIBC‐UAM) Universidad Autónoma de Madrid Madrid Spain

**Keywords:** Apiaceae, *Astydamia latifolia*, Canary Islands, islands colonization, phylogeography

## Abstract

*Astydamia latifolia* is the only species of the genus *Astydamia*, which forms an early‐diverging lineage of Apiaceae, subfamily Apioideae. This species is subendemic to the Canary Islands and one of the most representative species of the coastal environments of this archipelago. *Astydamia* displays diplochory, that is, diaspores with two long‐distance dispersal (LDD) syndromes. In particular, *A. latifolia* has both anemochorous and thalassochorous fruit traits (corky and winged mericarps). Although we expect this species to have a high dispersal capacity, there is no genetic study addressing it. The divergence time of this species from its sister taxon is also unknown. In this study, we aimed (i) to unveil the phylogenetic relationships and divergence times of *A. latifolia*; (ii) to reconstruct its phylogeographic structure across the Canary Islands; and (iii) to estimate the number of inter‐island colonization events. To these ends, we first sequenced the internal transcribed spacer (ITS) region for *A. latifolia*, reconstructed the phylogenetic relationships of *Astydamia* and Apiaceae relatives and estimated divergence times. Then, two plastid DNA regions (*psa*I‐*aac*D and *psb*K‐*trn*S) were sequenced for 158 individuals (from 36 Canarian population and one NW African population) to reconstruct a haplotype network. The recently developed method Phylogeographic Analysis of Island Colonization Events (PAICE) was used to estimate the number of inter‐island colonization events. Results show that *A. latifolia* is a phylogenetically isolated lineage that diverged from the most closely related genus (*Molopospermum*) in the Eocene–Miocene. It displays a low plastid DNA diversity (only four haplotypes detected), which is accompanied by a high degree of haplotype sharing between islands and highly linear rarefaction curves of colonization obtained in PAICE. These findings suggest a high colonization ability for this species, most likely related to the availability of two LDD syndromes.

## Graphical abstract

The iconic Canarian *Astydamia latifolia* is a diplochorous species. In this study, we inferred a high phylogenetic isolation of this species and recurrent inter‐island colonization across the Canarian archipelago, as highlighted by the new method PAICE.

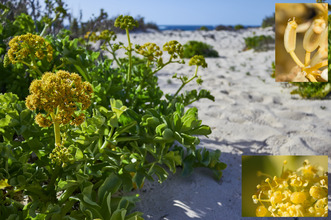

## 1 INTRODUCTION

Oceanic islands are among the most studied regions in evolutionary biology and biogeography, as they provide natural laboratories to test complex processes in a simplified setting (Vargas, 2014; Whittaker & Fernández‐Palacios, 2007). Following a biologically meaningful classification from Alfred R. Wallace, oceanic islands are frequently defined as land masses emerged from the oceanic floor typically as a result of volcanic activity (Ali, 2017). Because of this, oceanic islands have been completely isolated from other territories (e.g., continents) since their origin, and therefore terrestrial organisms occurring on them are necessarily the result of colonization from other land masses (Whittaker & Fernández‐Palacios, 2007).

Colonization is a two‐stage process in which (i) an organism or its diaspores travel from a source territory to a new area (dispersal), and then, (ii) this organism survives and reproduces in the new territory (establishment) (Heleno & Vargas, 2015; van der Pijl, 1982). Because of the sea barrier permanently separating oceanic islands from other land masses, long‐distance dispersal (LDD) is recognized as a fundamental process in their colonization (Nathan, 2006; Traveset et al., 2014). Therefore, it has been traditionally assumed that plant species with dispersal specializations in their fruits or seeds (known as dispersal syndromes; van der Pijl, 1982) are more successful in colonizing oceanic archipelagos than those with unspecialized diaspores (Carlquist, 1966). However, a more complex scenario has emerged in the last decade given the remarkably large proportion of species with unspecialized diaspores in archipelagos (Arjona et al., 2018; Heleno & Vargas, 2015; Nogales et al., 2012; Vargas et al., 2012).

The Canary Islands form an oceanic archipelago of Macaronesia, a group of archipelagos in the northeastern Atlantic Ocean mainly consisting of the Azores, Madeira, Cape Verde, and Canary Islands (Carracedo & Troll, 2021; Fernández‐Palacios et al., 2024). In particular, the Canary Islands are biogeographically related to the Mediterranean Basin, one of the 25 world hotspots of biodiversity described by Myers et al. (2000). It is also one of the best known archipelagos in the world from a floristic point of view (Emerson & Kolm, 2005), and a complete checklist of the Canarian flora is available (Acebes et al., 2010) and constantly updated (Biodiversity Data Bank of the Canary Islands, https://www.biodiversidadcanarias.es/biota/). This allowed categorization of angiosperm species of the Canary Islands into LDD syndromes by Vargas, Arjona, et al. (2015). Arjona et al. (2018) detected statistical support for dispersal and colonization advantages of Canarian species with LDD syndromes associated with their distribution within the archipelago (i.e., number of islands occupied).

*Astydamia latifolia* (L.f.) Baill. (Apiaceae) is a diplochorous species, as it displays two LDD syndromes, specifically thalassochorous and anemochorous syndromes (Alameda‐Martín, 2017; Vargas, Arjona, et al., 2015). This species occupies every major island of the Canarian archipelago (Acebes et al., 2010) and is also found in Northwest Africa (Dobignard & Chatelain, 2011; Médail & Quézel, 1999). *Astydamia latifolia* is the only species of its genus and it is part of an early‐diverging lineage of the *Annesorhiza* clade (tribe Annesorhizeae), in the Apiaceae family (Calviño et al., 2006, 2016; Downie et al., 2010; Magee et al., 2010, 2008; Nicolas & Plunkett, 2009). According to the lack of other species in the genus *Astydamia*, a high phylogenetic isolation and ancient divergence from its close living relatives is expected, as previously observed in other species of monotypic genera of Apiaceae such as *Naufraga balearica* Constance & Cannon (Fernández‐Mazuecos et al., 2014) and of other families such as *Gyrocaryum oppositifolium* Valdés (Boraginaceae; Otero et al., 2019) and *Drosophyllum lusitanicum* (L.) Link (Drosophyllaceae; Martín‐Rodríguez et al., 2020). This ancient divergence would hinder the estimation of a time frame for the colonization of the Canarian archipelago from the continent due to a possibly wide difference between stem and crown ages (Martín‐Hernanz et al., 2023). In any case, a high colonization ability is expected for *A. latifolia* because it displays two LDD syndromes (Alameda‐Martín, 2017; Arjona et al., 2018), a wide distribution in the Canarian archipelago (every major island) (Acebes et al., 2010), and no apparent habitat limitations (the coastal habitat occupied by *A. latifolia* is common on every island). To test this hypothesis, it would be necessary to quantify the number of inter‐island colonization events experienced by *A. latifolia* in the archipelago by analyzing the distribution of the genetic diversity and not only chorology (Coello et al., 2022; Vargas, Rumeu, et al., 2015). For this purpose, Coello et al. (2022) have recently proposed a novel method based on haplotype data and accounting for sample size, that is, the Phylogeographic Analysis of Island Colonization Events (PAICE). Additionally, the presence of *A. latifolia* populations in coastal Morocco is intriguing. It is unknown whether these African populations are derived from the ancestral continental range from which the Canary Islands were colonized or, alternatively, African populations are the result of back‐colonization from the archipelago to the continent.

In this study, we aimed to estimate divergence times based on nuclear internal transcribed spacer (ITS) sequences and to unveil phylogeographic patterns of *A. latifolia* based on plastid DNA (cpDNA). We hypothesized that *A. latifolia*, as the only species of its genus, diverged a long time ago from its closest living relatives. Furthermore, we aimed to estimate the dispersal capacity of this species by assessing its genetic diversity in the Canary Islands and Morocco and the number of inter‐island colonization events. As *A. latifolia* is a diplochorous species, we hypothesized that this species displays a high colonization capacity. In particular, our specific objectives were (i) to infer the divergence time between *A. latifolia* and its closest living relatives, (ii) to describe the geographic distribution of the plastid DNA diversity of *A. latifolia* in the Canary Islands and Morocco, and (iii) to estimate the number of inter‐island colonization events using PAICE.

## 2 MATERIALS AND METHODS

### 2.1 Study species

**FIGURE 1:**
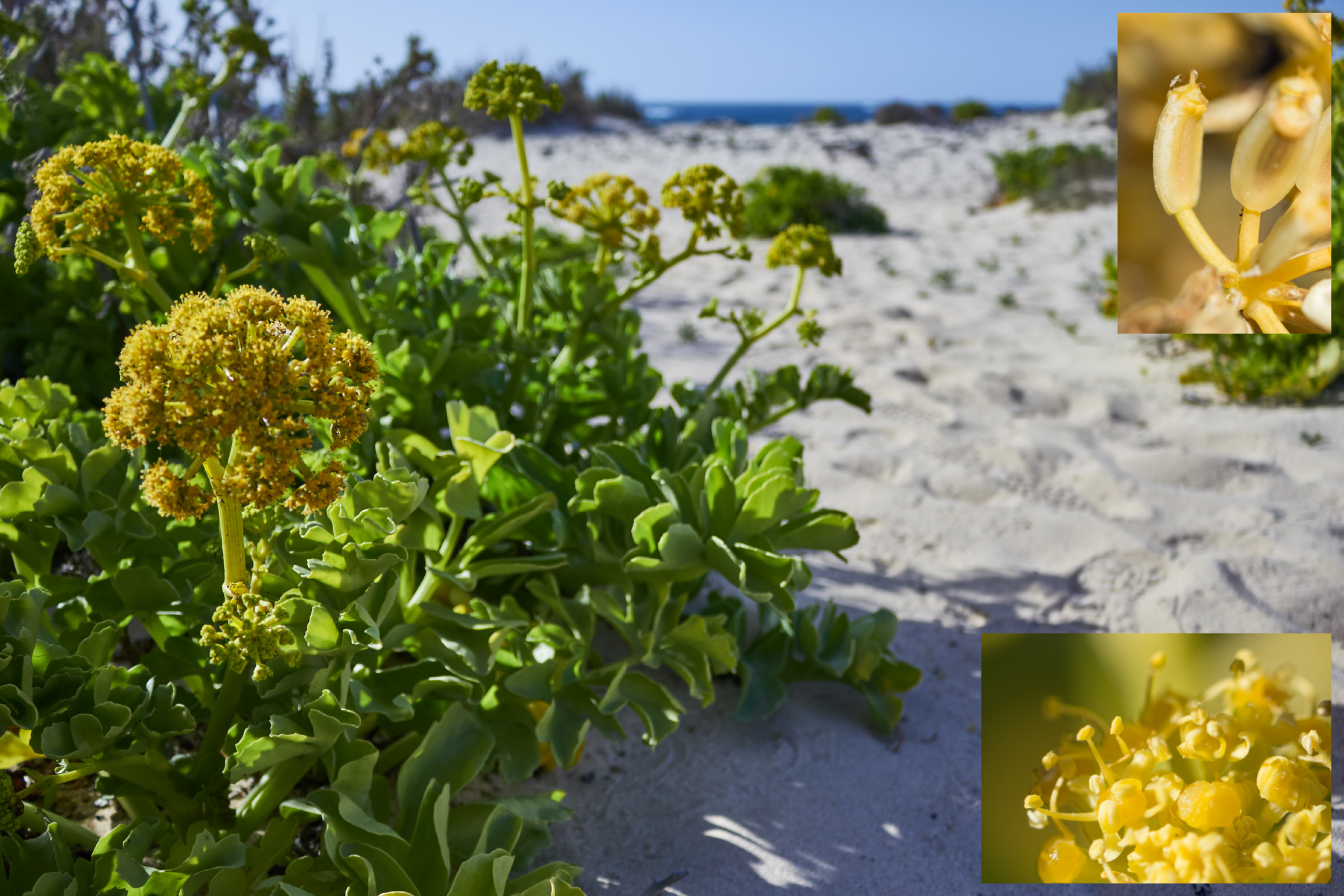
*Astyamia latifolia* in El Cotillo (Fuerteventura, Canary Islands). Insets show fruit (top) and flower (bottom) details in individuals from Punta de la Calera (La Gomera). Photos taken by Alberto J. Coello.

*Astydamia latifolia* (Figure 1) is the only species of the genus *Astydamia* DC., which belongs to the Annesorhizeae tribe of Apiaceae according to molecular phylogenetic data (Calviño et al., 2006, 2016; Downie et al., 2010; Magee et al., 2008; Nicolas & Plunkett, 2009). The genera most closely related to *Astydamia* are *Molopospermum* W.D.J.Koch and *Ezosciadium* B.L.Burtt, and these three genera form a clade sister to all remaining Annesorhizeae (Calviño et al., 2006, 2016; Magee et al., 2008). However, the divergence time between *Astydamia* and its sister genus is still unknown. Morphologically, *A. latifolia* is a succulent hemicryptophyte less than 50 cm tall subendemic to the Canary Islands, with additional localities in the Saharan coast and Savage Islands (Acebes et al., 2010; Bramwell & Bramwell, 2001; Dobignard & Chatelain, 2011; Fennane, 2017; GBIF.org, 2021; Médail & Quézel, 1999). *Astydamia latifolia* is a salinity‐resistant species that grows on dunes, beaches, rocks, and cliffs in coastal habitats.

The oceanic archipelago of the Canary Islands is located between 27.5°–29.5°N and 13°–18.5°W, less than 100 km from Africa. The seven largest islands of the archipelago are inhabited by *A. latifolia* (although this species is less frequent in eastern islands), from east (oldest) to west (youngest): Lanzarote, Fuerteventura, Gran Canaria, Tenerife, La Gomera, La Palma, and El Hierro (Troll & Carracedo, 2016). According to LDD syndrome categorization, *A. latifolia* is a diplochorous species combining anemochorous and thalassochorous LDD syndromes (Alameda‐Martín, 2017; Arjona et al., 2018; Vargas, Arjona, et al., 2015). This is because it displays winged and corky mericarps, and its seeds are able to survive on seawater. In fact, it has been observed that more than 50% of *A. latifolia* seeds remain viable after floating on seawater for a long period of time (i.e., more than 7 days; Alameda‐Martín, 2017; Bramwell, 1985).

### 2.2 Sampling and DNA sequencing

We obtained fresh leaves of 155 individuals of *A. latifolia* from 36 Canarian populations (up to five individuals per population) and three individuals from the Saharan population (Table S1). All materials collected in the field were dried in silica gel until DNA extraction. We also used two herbarium specimens of *Molopospermum peloponnesiacum* (L.) W.D.J.Koch (from the MA herbarium) as outgroup for the phylogeographic analysis given the close relationship between *Molopospermum* and *Astydamia* (Table S1) (Calviño et al., 2006; Magee et al., 2008). We extracted total genomic DNA using the DNeasy Plant Kit (QIAGEN Inc., California) following the manufacturer's instructions. For phylogenetic analysis (see below), we sequenced the ITS region (Table 1) for a representative sample of three Canarian *A. latifolia* individuals and the two samples of *M. peloponessiacum*. To select variable cpDNA regions for phylogeographic analysis (see below), we performed a pilot study using 19 regions (Alameda‐Martín, 2017 and this study) included among the most variable regions of the plastid genome of angiosperms (Shaw et al., 2007). As a result, we selected the two most variable cpDNA regions: *psa*I‐*aac*D and *psb*K‐*trn*S (Table 1). Each DNA region was amplified by conventional PCR in an Eppendorf Mastercycler EPGradient S (Eppendorf GmbH, Hamburg, Germany) following these conditions: after 2 min of pretreatment at 94°C, we conducted 30 cycles of 94°C for 1 min, 52°C for 1 min and 72°C for 1 min, followed by a final elongation period of 10 min at 72°C. We added 1 μL of bovine serum albumin at 1 mg/mL to every 25 mL of reaction to improve the amplification efficiency. PCR products were stored at 4°C until their Sanger sequencing by Macrogen Inc. (Madrid, Spain). For each sequence, we assembled forward and reverse electropherograms in Geneious v11.0.4 (Kearse et al., 2012).

**TABLE 1:** Primers used for amplification and sequencing of DNA regions in *Astydamia latifolia* and outgroup species.

Region	Primer sequences	Reference
ITS	ITS5: GGA AGT AAA AGT CGT AAC AAG G ITS4: TCC TCC GCT TAT TGA TAT GC	White et al. (1990)
*psa*I‐*aac*D	psaI: AGA AGC CAT TGC AAT TGC CGG AAA aacD: AAT YGT ACC ACG TAA TCY TTT AAA	Shaw et al. (2007)
*psb*K‐*trn*S	psbK for: GCC TTT GTT TGG CAA GCT GC trnS rev: CGA GTT ATT CGT ACC GAG GG	Fernández‐Mazuecos and Vargas (2010)

### 2.3 Phylogenetic analysis and divergence times

**FIGURE 2:**
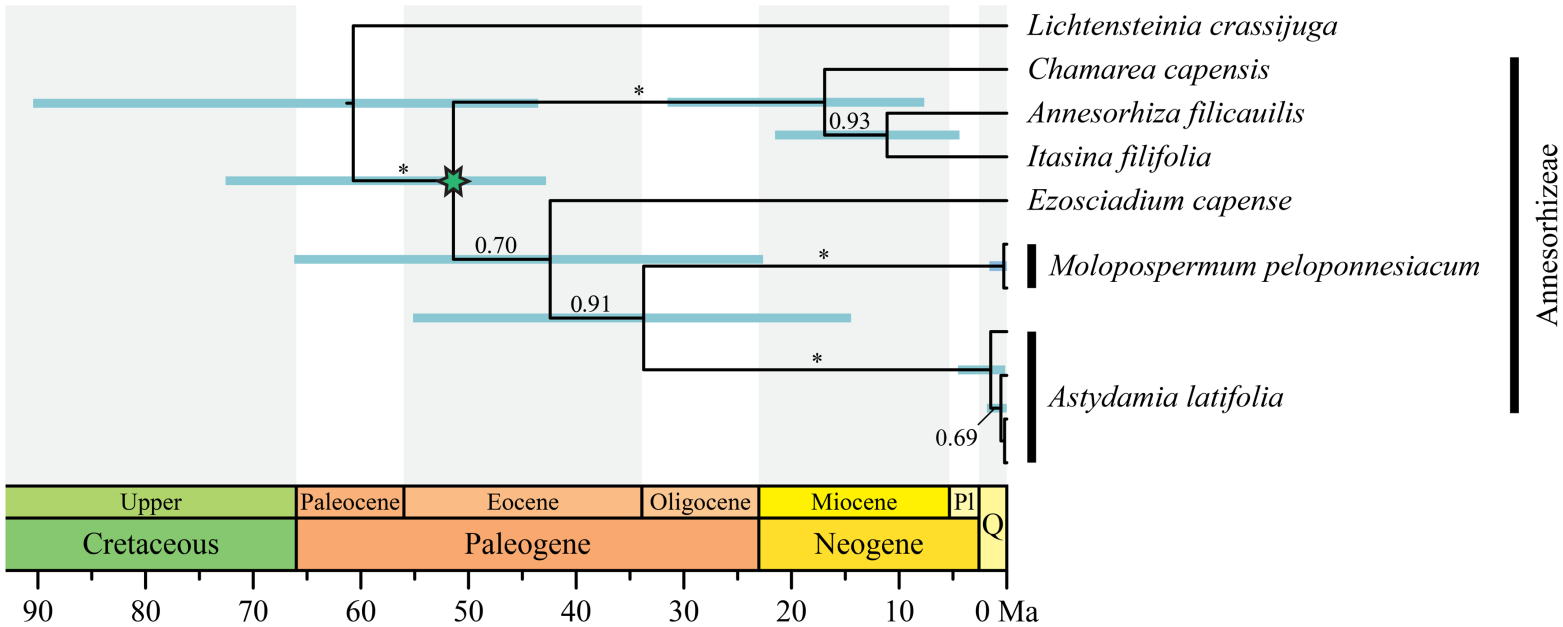
Time‐calibrated Bayesian phylogenetic analysis of *Astydamia latifolia* and relatives based on the ITS region. The maximum clade credibility tree is shown. Posterior probability values (PP) are shown above branches when PP >0.5, and asterisks (*) indicate PP ≥0.95. The 95% highest posterior density intervals for node ages are shown when PP ≥0.5. The green star indicates the calibration point (see text).

To test the monophyly of the populations of *A. latifolia* and to estimate the divergence time between this species and its living relatives, we performed a time‐calibrated phylogenetic analysis of the tribe Annesorhizeae based on ITS sequences, with the genus *Lichtensteinia* as the outgroup. More distantly related lineages were not included given the high divergence of ITS sequences in Apiaceae (Calviño et al., 2006; Calviño & Downie, 2007; Downie et al., 2010). We downloaded ITS sequences for Annesorhizeae genera and *Lichtensteinia* from Genbank (Table S2) and they were aligned together with our new ITS sequences using MAFFT v7.490 (Katoh et al., 2002). This ITS alignment (Alignment 1 in Appendix S1) was used to perform a Bayesian time‐calibrated phylogenetic analysis in BEAST v1.8.4 (Drummond & Rambaut, 2007). A secondary calibration was implemented for the time of divergence between *Lichtensteinia* and Annesorhizeae, corresponding to the crown node of Apioideae, with a uniform distribution from 45.9 to 76.1 Ma following the results of Calviño et al. (2016) (Figure 2). In addition, Annesorhizeae was constrained to be monophyletic (Calviño et al., 2016; Downie et al., 2010). We applied a GTR + G model of nucleotide substitution, as it was the best‐fitting model estimated by the AIC criterion in jModelTest v2.1.10 (Darriba et al., 2012). An uncorrelated relaxed clock with a lognormal distribution was implemented, and a birth‐death speciation process was defined as tree prior. We executed two runs with 10 million generations each, sampled every 1000 generations, and applied a 10% burn‐in. Convergence between runs was confirmed in Tracer v1.7.1 (Rambaut et al., 2018). Then, both runs were combined using LogCombiner (discarding the burn‐in) and trees were summarized in a maximum clade credibility tree with median node heights in TreeAnnotator.

### 2.4 Phylogeographic analysis

To describe the geographic distribution of the plastid DNA diversity of *A. latifolia*, we used the *psa*I‐*aac*D and *psb*K‐*trn*S regions (Table S1). As outgroup, we used the two samples of *Molopospermum peloponnesiacum* (Table S1). We aligned both regions with MAFFT v7.388 (Katoh et al., 2002). Alignments were manually edited, and we coded an inversion of three bases in the *psa*I‐*aac*D region (positions 133–135) as a single mutational step. The two cpDNA regions were concatenated in a single alignment and used to determine the relationships among haplotypes of *A. latifolia* (Alignment 2 in Appendix S2). We used the statistical parsimony algorithm (Templeton et al., 1992) implemented in TCS v.1.21 (Clement et al., 2000), considering a 95% confidence limit and gaps as missing data. We also used the haplotypes detected by TCS to calculate the number of haplotypes (*h*), the number of private haplotypes (*ph*), and the haplotypic diversity (*H*; calculated as indicated in Nei & Tajima, 1981).

### 2.5 Estimation of inter‐island colonization events

We inferred the minimum number of colonization events between islands of the Canarian archipelago with the *colonization* function of the R package PAICE (Data S1 and Script 1 in Appendix S3; Coello et al., 2022). We considered Lanzarote and Fuerteventura as a single island called Mahan because sea level oscillations connected Lanzarote and Fuerteventura during the Last Glacial Maximum (Troll & Carracedo, 2016). With this strategy, we avoided confounding inter‐island colonization events and land diffusion.

Given that the inference of the minimum number of colonization events using only haplotype distribution ranges is highly biased by sampling effort (Coello et al., 2021, 2022), we also calculated asymptotic estimators of inter‐island colonization events as implemented in PAICE (Data S1 and Script 1 in Appendix S3; Coello et al., 2022). To calculate the minimum number of inter‐island colonization events and estimate asymptotic estimators, we excluded the Moroccan population (because we were only interested in estimating colonization events within the Canarian archipelago). We used the *rarecol* function to generate rarefaction curves replicated a number of times equal to five times the number of levels for each sampling variable, that is, 180 replicates for field sampling (36 levels, one for each of the 36 populations sampled) and 25 replicates for genetic sampling (five levels corresponding to the four variable positions observed in the alignment plus the case of no genetic information available, i.e., considering only the number of islands occupied by the species). Rarefaction curves were used to calculate the asymptotic estimators of inter‐island colonization events using *maxCol*, deleting the 5% extreme values (i.e., argument *del* = 0.05) and calculating the 95% confidence interval of asymptotic estimators (i.e., argument *level* = 0.95). We also set up argument *method* = 1 to allow the algorithm to fit accumulation curves of colonization with fewer parameters in cases in which curves could not be fitted with all parameters. As *M. peloponnesiacum* was not connected to *A. latifolia* in the haplotype network (see Section 3), we could not confidently assign the ancestral haplotype. To resolve this, we performed this analysis four times, each of them considering a different haplotype as hypothetical ancestral haplotype in the archipelago.

## 3 RESULTS

### 3.1 Phylogenetic relationships and divergence times of *Astydamia latifolia*

The time‐calibrated Bayesian analysis of the ITS region for Annesorhizeae (651 bp, Alignment 1 in Appendix S1) is shown in Figure 2. It displays *A. latifolia* as a monophyletic group (PP = 1) sister to *Molopospermum peloponnesiacum* (PP = 0.91). The divergence between both species (stem age of *A. latifolia*) was estimated to have happened in the Eocene – Miocene, with a mean of 33.73 Ma (95% highest posterior density 14.47–55.15 Ma), while a Pliocene–Pleistocene crown age of *A. latifolia* was estimated, with a mean of 1.49 Ma (0.15–4.54 Ma). This phylogenetic reconstruction also recovered a separate monophyletic group with three genera (*Annesorhiza* Cham. & Schltdl, *Chamarea* Eckl. & Zeyh. and *Itasina* Raf.) within Annesorhizeae (PP = 1), while the phylogenetic position of *Ezosciadium* had low support (PP = 0.70 for a clade including *Ezosciadium*, *Molopospermum* and *Astydamia*).

### 3.2 Phylogeographic analysis of *Astydamia latifolia*

**FIGURE 3:**
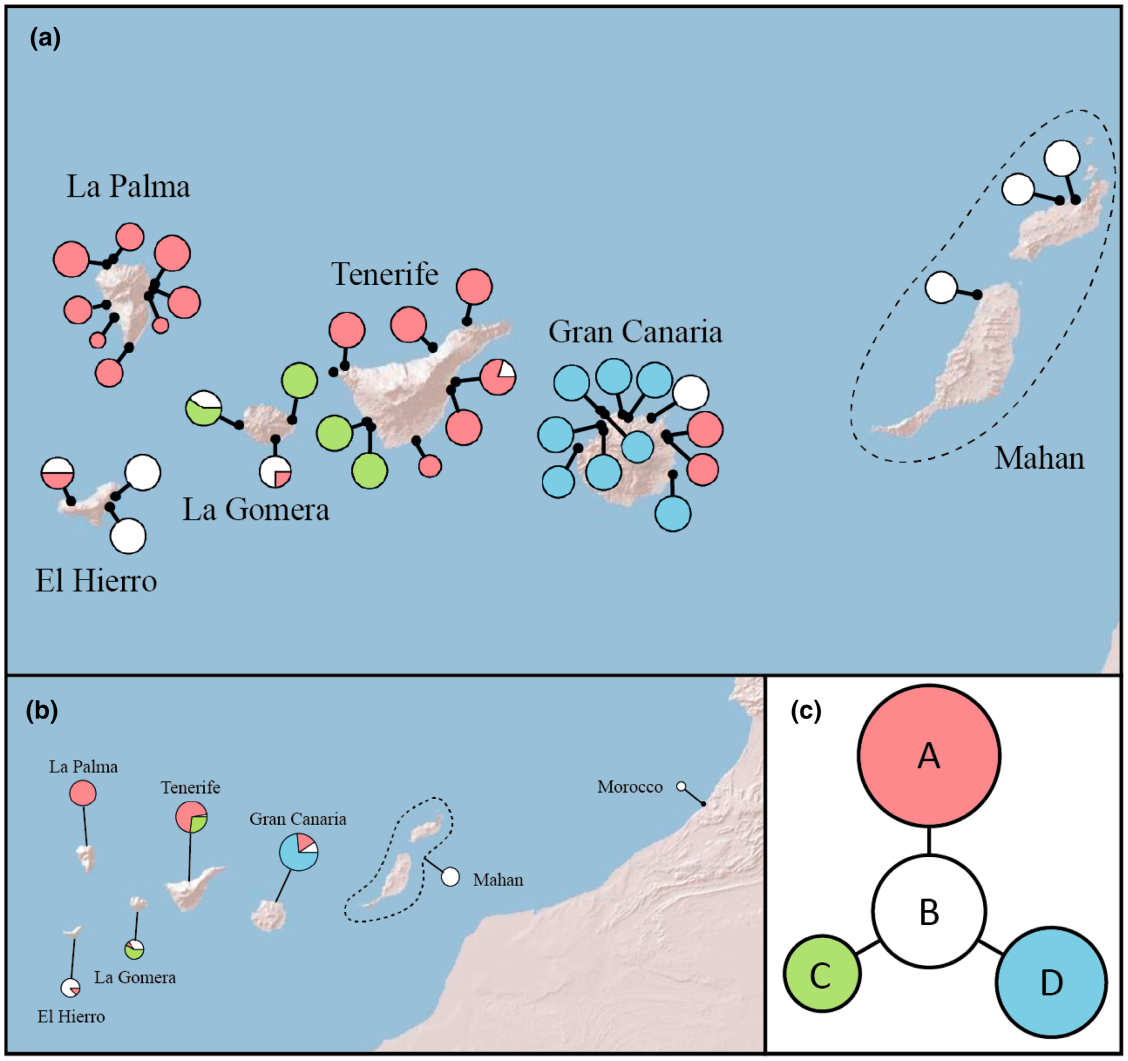
Phylogeographic reconstruction of *Astydamia latifolia* using two cpDNA regions: *psa*I‐*aac*D and *psb*K‐*trn*S. (a) Haplotype frequencies in sampled populations of the Canary Islands. (b) General view of haplotype distribution in the Canary Islands (summarized by island and considering Lanzarote and Fuerteventura as a single paleoisland called Mahan) and Morocco. (c) Genealogical relationships among *A. latifolia* haplotypes; note that the two individuals (haplotypes) of *Molopospermum peloponnesiacum* were not connected to *A. latifolia* haplotypes. Circle sizes are proportional to haplotype frequencies.

We managed to sequence 158 individuals of *A. latifolia* from 36 Canarian populations and one Moroccan population for the two cpDNA regions: *psa*I‐*aac*D and *psb*K‐*trn*S (Table S1). Two individuals of *M. peloponnesiacum* from the MA herbarium were fully sequenced for the two cpDNA regions. The *psa*I‐*aac*D and *psb*K‐*trn*S regions resulted in alignments of 792 bp (first part of Alignment 2 in Appendix S2) and 705 bp respectively (second part of Alignment 2 in Appendix S2). Therefore, the concatenated alignment had 1497 bp (Alignment 2 in Appendix S2). For *psa*I‐*aac*D, we coded an inversion in positions 133–135 as a single mutation step. The TCS analysis recovered six haplotypes, two of which corresponded to *M. peloponnesiacum* and four to *A. latifolia* (Figure 3), but both species formed disconnected haplotype networks. The two *M. peloponnesiacum* haplotypes were connected by a single mutation step (data not shown). In the *A. latifolia* network, haplotypes A, C and D were all directly connected to central haplotype B by a single mutation step. Haplotype A was the most common (40% of *A. latifolia* individuals), and it was distributed on the five central and western islands of the Canarian archipelago (all except Lanzarote and Fuerteventura). Haplotypes B and D displayed similar frequencies (23% and 25% respectively), but the central haplotype B had a wider distribution (it occurred on every major island of the Canarian archipelago except for La Palma, and it was also found in the Moroccan population). Meanwhile, haplotype D was only found in Gran Canaria, and haplotype C was present in southwestern Tenerife and La Gomera. The three central islands (La Gomera, Tenerife and Gran Canaria) harbored the highest number of haplotypes (three haplotypes on each island) and La Gomera was the island with the highest haplotypic diversity (Table 2).

**TABLE 2:** Haplotypic diversity of *Astydamia latifolia* in the Canary Islands and Morocco based on two cpDNA regions (*psa*I‐*aac*D and *psb*K‐*trn*S).

Area	*n*	*p*	*h*	*ph*	*H*
Morocco	3	1	1	0	0
Mahan (Lanzarote + Fuerteventura)	13	3	1	0	0
Gran Canaria	52	11	3	1	0.435
Tenerife	37	9	3	0	0.444
La Gomera	14	3	3	0	0.582
La Palma	25	8	1	0	0
El Hierro	14	3	2	0	0.264

Abbreviations: *h*, number of haplotypes; *H*, haplotypic diversity; *n*, number of individuals sequenced; *p*, number of populations; *ph*, private haplotypes.

### 3.3 Inter‐island colonization events

**FIGURE 4:**
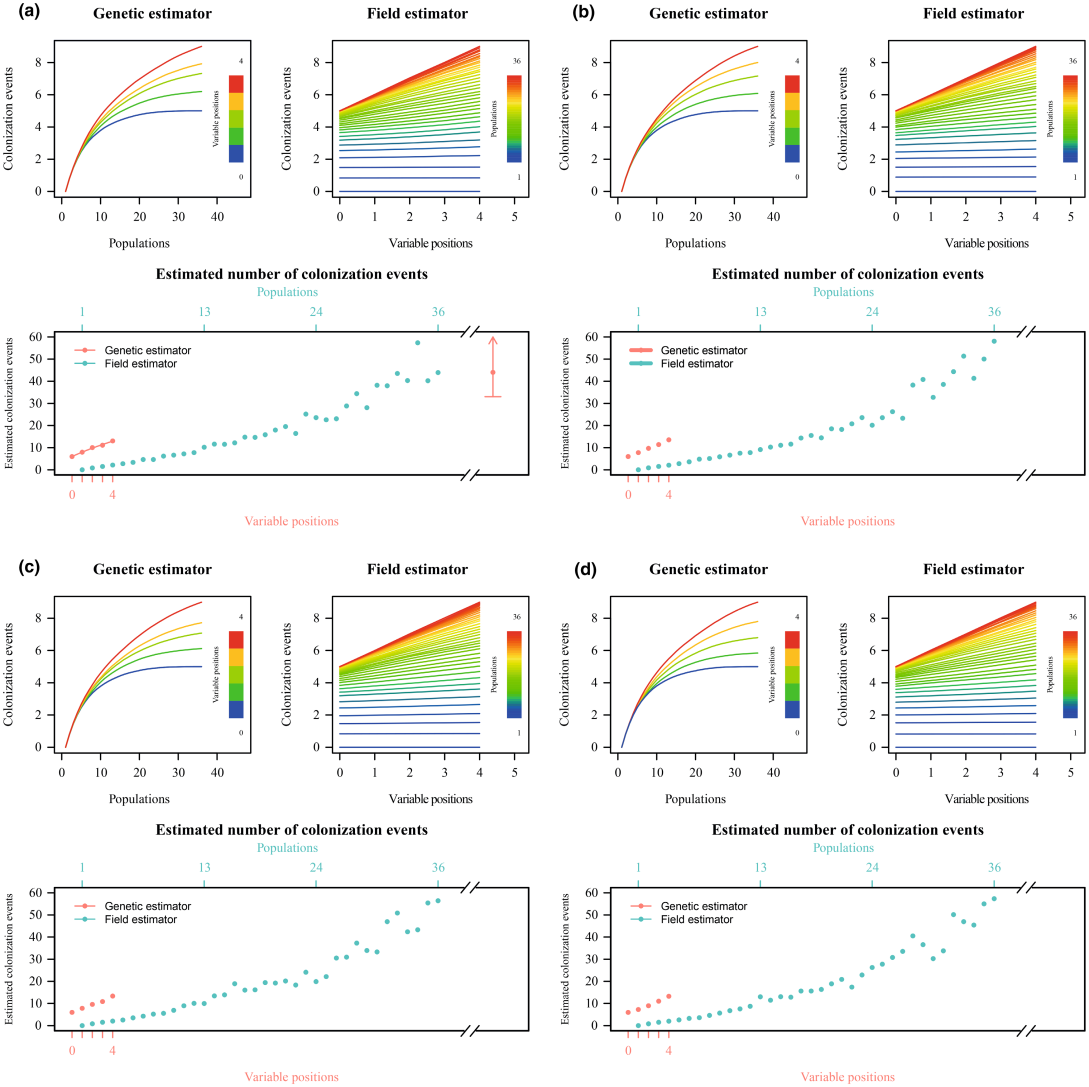
Estimation of inter‐island colonization events of *Astydamia latifolia* across the Canarian archipelago according to PAICE, which corrects for sampling effort. In each panel, the two top plots represent rarefaction curves of colonization events considering genetic and field estimator methods respectively, and the bottom plot represents the estimation of the number of inter‐island colonization events corrected for sampling effort; dots represent the mean value and bars indicate the 95% confidence intervals (an arrow indicates that the upper limit is infinite). Different scenarios were considered because the outgroup is disconnected from Canary Island haplotypes; in each case the ancestral haplotype considered was (a) haplotype A, (b) haplotype B, (c) haplotype C, and (d) haplotype D.

According to PAICE results, the geographic distribution of the diversity of *A. latifolia* haplotypes in the Canary Islands was explained by a minimum of nine inter‐island colonization events. When considering sampling size in the estimation of inter‐island colonization events (Figure 4), it was only possible to estimate the asymptotic estimator of inter‐island colonization events for the genetic estimator when haplotype A was considered as the ancestral haplotype in the archipelago. In this case, 44.0 colonization events were estimated (95% confidence interval 33.0–∞ colonization events). In the rest of cases (i.e., field estimator when using haplotype A as ancestral haplotype, and both estimators when haplotypes B, C, or D were considered as the ancestral haplotype) it was impossible to calculate asymptotic estimators.

## 4 DISCUSSION

In this study, we confirmed the extreme phylogenetic isolation of the monotypic genus *Astydamia*, as the divergence from its closest living relative (*Molopospermum*) dates back to around the Eocene–Miocene, c. 33.73 Ma (14.47–55.15 Ma). The lack of resolution among *A. latifolia* haplotypes prevented the identification of Saharan populations as either the continental source of colonization of the Canarian archipelago or the result of a recent back‐colonization of the continent from the islands. Despite the limited plastid DNA variation detected across Canarian populations, PAICE results suggested a large number of inter‐island colonization events. Potential causes for successful colonization are discussed below.

### 4.1 A phylogenetically singular lineage

Our phylogenetic reconstruction based on ITS sequences (Figure 2) suggests the presence of two clades in the tribe Annesorhizeae, in agreement with Calviño et al. (2016): a strongly supported clade including *Chamarea*, *Annesorhiza* and *Itasina* (PP = 1); and a poorly supported clade including *Ezosciadium*, *Molopospermum* and *Astydamia* (PP = 0.70). Results within the latter clade strongly support the monophyly of *Astydamia* and indicate a close relationship between *Astydamia* and *Molopospermum* (PP = 0.91; Figure 2), as reported in previous studies (Calviño et al., 2006, 2016).

Focusing on *A. latifolia*, the estimated age of the most recent common ancestor of all extant populations is recent (around the Pliocene–Pleistocene, c. 1.49 Ma; 95% HPD = 0.15–4.54 Ma), while the divergence between *Astydamia* and *Molopospermum* appears to be much older (around the Eocene–Miocene, c. 33.73 Ma; 95% HPD = 14.47–55.15 Ma) (Figure 2). This long interval between the stem and crown age of *A. latifolia* highlights the great phylogenetic singularity of this species, similar to that found for *Drosophyllum lusitanicum* (Martín‐Rodríguez et al., 2020), *Gyrocaryum oppositifolium* (Otero et al., 2019) or *Naufraga balearica* (Fernández‐Mazuecos et al., 2014). This phylogenetic singularity (Figure 2) may be the result of either low speciation or high extinction in the *Astydamia* lineage. The old divergence from the closest relatives and the low number of haplotypes observed among *A. latifolia* populations (Figure 3) suggests a recent biogeographic connection between Canarian and African populations. One possibility is to interpret African populations as a recent continental source of island colonization. Based on time‐calibrated phylogenetic results, the arrival of *A. latifolia* in the Canarian archipelago may have happened at any time between its differentiation from *Molopospermum* in the Eocene–Miocene and the split of extant populations in the Pliocene–Pleistocene (García‐Verdugo, Caujapé‐Castells, & Sanmartín, 2019; Martín‐Hernanz et al., 2023). Although the oldest currently emerged islands of the Canarian archipelago (Lanzarote and Fuerteventura) date back to c. 25 Ma, other paleoislands emerged up to c. 68 Ma (Troll & Carracedo, 2016). Alternatively, it is possible that *A. latifolia* is an example of back‐colonization, a relative infrequent pattern in which an insular lineage goes back to the continent, with some examples among plant lineages that are shared between the Canary Islands and the Mediterranean Region (e.g., Carine et al., 2004; García‐Verdugo et al., 2021; Jaén‐Molina et al., 2009, 2021). In the particular case of *A. latifolia*, our results (Figure 3) are compatible with the Saharan populations being the colonization source from the continent to the archipelago. This is because haplotype B is the most probable ancestral haplotype, as it is the central haplotype in the network (the haplotype with a higher number of connections) and it is widely distributed across the archipelago. However, more variable molecular markers and an extended sample are needed to test this hypothesis.

### 4.2 Plastid genetic structure of Canarian populations

The relatively low cpDNA diversity in Canarian populations of *A. latifolia* (Figure 3) is remarkable considering the high isolation of the *A. latifolia* lineage from its closest living relative (Figure 2). Only four haplotypes were detected using the two most variable cpDNA regions, and two of these haplotypes (A and B) are found on at least five islands (haplotype B was also detected in the Moroccan population). A pattern of higher haplotypic diversity was found in central islands (La Gomera, Tenerife, Gran Canaria), among which La Gomera had the highest value (Table 2). This pattern of highest genetic diversity in central islands has also been detected in other Canarian plants; for example, *Canarina canariensis* (L.) Vatke (Mairal et al., 2015), *Cistus monspeliensis* L. (Coello et al., 2021; Fernández‐Mazuecos & Vargas, 2011), *Euphorbia canariensis* L. (Coello et al., 2023), and *Periploca laevigata* Aiton (García‐Verdugo et al., 2017). In fact, the low genetic diversity of easternmost islands can be related to the different environment of Lanzarote and Fuerteventura compared to the rest of the archipelago (del Arco Aguilar & Rodríguez Delgado, 2018). Indeed, the relatively rare presence of *A. latifolia* in Lanzarote and Fuerteventura and the observation of a single haplotype suggest that these islands have been colonized recently, as found for other Canarian species (García‐Verdugo, Caujapé‐Castells, Illera, et al., 2019). However, an alternative relictual explanation is also possible, according to which populations in easternmost islands would be remnants of ancestral populations, and their lack of genetic diversity could be associated with an intense bottleneck, as observed for *Cistus monspeliensis* in the Mediterranean region (Coello et al., 2021).

### 4.3 High colonization success of *Astydamia latifolia*

Considering the low phylogeographic structure of Canarian populations, with several islands sharing the same haplotypes (Figure 3), the colonization capacity of *A. latifolia* seems to be very high, as it has been previously suggested (Alameda‐Martín, 2017). The predominant maternal inheritance of cpDNA in angiosperms (Corriveau & Coleman, 1988) led us to interpret that the pattern of haplotype distribution shows the footprint of seed movements within the archipelago. Therefore, sharing of haplotypes among several islands is the result of numerous inter‐island colonization events (Coello et al., 2022; Vargas, Rumeu, et al., 2015).

It is generally assumed that species of oceanic archipelagos follow the so‐called “progression rule,” according to which the oldest emerged islands were colonized first, and the subsequent colonization pattern of the archipelago is congruent with the emergence times of islands (Shaw & Gillespie, 2016). Although this pattern is widely observed, including in the Canary Islands (e.g., Villa‐Machío et al., 2020), there are numerous examples in the literature in which the colonization pattern of an archipelago is more complex (e.g., Fernández‐Mazuecos et al., 2020). In the particular case of *A. latifolia*, colonization between islands of the Canarian archipelago appears to have been recurrent, as shown by haplotype sharing among islands (Figure 3). Indeed, at least nine inter‐island colonization events are needed to explain the geographic distribution of haplotypes among the six major islands (as Lanzarote and Fuerteventura are considered together as a single paleoisland called Mahan; Troll & Carracedo, 2016). However, our attempt to correct for sampling size (Coello et al., 2022) resulted in an almost linear accumulation of colonization events in rarefaction curves (Figure 4). In particular, rarefaction curves lacked any curvature when they were constructed as a function of genetic sampling (field estimator rarefaction curves, Figure 4), suggesting that genetic information hides many colonization events, in a similar way as observed for *Xylocopa darwini* Cockerell, 1926 in the Galápagos (Coello et al., 2022). In these cases, the use of more informative sequencing techniques (i.e., next‐generation sequencing) is highly recommended (e.g., Fernández‐Mazuecos et al., 2018; Gallego‐Narbón et al., 2022). In particular, if plastid genomes were completely sequenced, the effect of genetic sampling effort would be reduced drastically and then, rarefaction curves implemented in PAICE (Coello et al., 2022) could be generated only for field sampling to obtain the final estimators. This would improve the accuracy of estimations and reduce computing time. In fact, other techniques to estimate the number of inter‐island colonization events could not be applied due to the low genetic variability of Canarian population. In particular, a Discrete Phylogeographic Analysis (Lemey et al., 2009) did not reach convergence after 1000 million generations of analysis (data not shown).

In any case, the number of colonization events for *A. latifolia* in the Canary Islands seems to be very high considering the low curvature of rarefaction curves (Figure 4). This estimate of inter‐island colonization events, resulting from the considerable sharing of haplotypes among islands, is congruent with the two dispersal syndromes shown by *A. latifolia* fruits: thalassochorous and anemochorous (Alameda‐Martín, 2017; Arjona et al., 2018). Previously, it was shown that the number of islands occupied by diplochorous species is not necessarily greater than that observed for monochorous species (Vargas, Arjona, et al., 2015). However, considering not only the chorology (number of islands inhabited) but also the number of inter‐island colonization events, *A. latifolia* appears to have a very high colonization ability. In particular, *A. latifolia* displays an efficient thalassochorous syndrome, as its fruits have structures that favor floatability on sea water and its seed are able to germinate after several days of transport across the sea (Alameda‐Martín, 2017). In this regard, *A. latifolia* seems to be similar to *Cryptocarpus pyriformis* Kunth (Nyctaginaceae), a thalassochorous species from the Galápagos with high dispersal ability (Arjona et al., 2020). It is logical to suppose that the inter‐island connectivity of *A. latifolia* populations was favored by sea currents, as found for *C. pyriformis*. In fact, the presence of haplotype C only in southwestern Tenerife and La Gomera could be related to whirlpools between these islands, while the distribution of haplotype B seems to be congruent with a NE–SW current (Figure 3; see Martínez et al., 2024). However, these hypotheses require further study (e.g., Arjona et al., 2020) and the additional consideration of winds, given the presence of both thalassochorous and anemochorous syndromes in *A. latifolia* (Vargas, Arjona, et al., 2015).

## 5 CONCLUSIONS

*Astydamia latifolia* (the only species of the genus *Astydamia*) displayed a high phylogenetic isolation after an early divergence, in the Eocene – Miocene, from its sister lineage (*Molopospermum*). In contrast, we observed low cpDNA variation for *Astydamia latifolia* in the Canary Islands and a lack of genetic differentiation with respect to mainland African populations. The identification of the mainland population as either ancestral or the result of a back‐colonization from the Canary Islands was not possible with our data. Furthermore, the low phylogeographic structure of Canarian populations suggested a high colonization ability for *A. latifolia*, corroborated by PAICE analysis, specifically by the linear tendency of rarefaction curves of colonization events as a function of genetic sampling, and related to the two LDD syndromes displayed by the diaspores of *A. latifolia*.

In future studies, an increase in genetic sample size will be needed to corroborate the high colonization ability of *A. latifolia*, and an increase in field sample size in Northwest Africa and Savage Islands will be required to untangle the relationships between island and mainland populations. Furthermore, additional analyses are needed to understand factors related to the colonization potential of this species, such as habitat availability responsible for establishment (Heleno & Vargas, 2015; van der Pijl, 1982), and ocean currents affecting the Canary Islands and potentially explaining movement and genetic connection between populations (Arjona et al., 2020).

## AUTHOR CONTRIBUTIONS

**Alberto J. Coello:** Conceptualization (equal); methodology (equal); writing – original draft (equal); writing – review and editing (equal). **Pablo Vargas:** Conceptualization (equal); funding acquisition (lead); writing – review and editing (equal). **Aitor Alameda‐Martín:** Methodology (equal); writing – review and editing (equal). **Emilio Cano:** Methodology (equal); writing – review and editing (equal). **Yurena Arjona:** Methodology (equal); writing – review and editing (equal). **Mario Fernández‐Mazuecos:** Conceptualization (equal); methodology (equal); writing – original draft (equal); writing – review and editing (equal).

## FUNDING INFORMATION

This study is part of the projects CGL2015‐67865‐P and PGC2018‐101650‐B‐I00, funded by the Spanish Ministry of Economy, Industry and Competitiveness. A.J.C. was supported by the Spanish Ministry of Education, Culture and Sport through an FPU fellowship (FPU16/05681). M.F.‐M. was supported by the Spanish Ministry of Economy and Competitiveness through a Juan de la Cierva fellowship (IJCI‐2015‐23,459), and by the Spanish National Research Council (CSIC) through a Special Intramural Project (201930E078).

## CONFLICT OF INTEREST STATEMENT

The authors declare no conflicts of interest.

## Supporting information


Tables S1–S2

Data S1

Appendix S1

Appendix S2

Appendix S3

## Data Availability

DNA sequences are available in GenBank (accession numbers are shown in Tables S1 and S2). Sequence alignments for both ITS (Alignment 1 in Appendix S1) and plastid DNA (*psa*I‐*aac*D and *psb*K‐*trn*S; Alignment 2 in Appendix S2) are available in the Supporting Information. The script for the estimation of inter‐island colonization events (Script 1 in Appendix S3) and the data to perform the analysis (Data S1) are available in the Supporting Information.
