# Supplementary material for: Phylogenetic and phylogeographic evidence for the colonization success of the diplochorous Astydamia latifolia across the Canary Islands
Source: Ecol Evol. 2024 Jul 4;14(7):e11624. doi: 10.1002/ece3.11624 (PMC11222747; doi:10.1002/ece3.11624)
Supplement: Supplementary file 1 — Tables S1–S2 [file ECE3-14-e11624-s005.pdf]

Table S1: Samples of *Astydamia latifolia* and *Molopospermum peloponnesiacum* used in this study. Locality information (with coordinates), vouchers and haplotypes of *A. latifolia* detected for plastid regions are indicated.

Taxon	Locality (coordinates)	Voucher	Haplotypes	Accession numbers<br>*psa*I-*aac*D	 *psb*K-*trn*S
Astydamia latifolia	Morocco, Tiznit, Ifni Mesti (29.346N, 10.196W)	E. Rico DG497	B	PP803867, PP803868, PP803869	PP804028, PP804029, PP804030
	Spain, El Hierro, Puerto de la Estaca (27.780N, 17.906W)	A. J. Coello & M. Fernández-Mazuecos 40AC18	B	PP803748, PP803749, PP803750, PP803751, PP803752	PP803909, PP803910, PP803911, PP803912, PP803913
	Spain, El Hierro, Punta del Guincho (27.731N, 17.930W)	A. J. Coello & M. Fernández-Mazuecos 36AC18	B	PP803753, PP803754, PP803755, PP803756, PP803757	PP803914, PP803915, PP803916, PP803917, PP803918
	Spain, El Hierro, Punta Gorda (27.756N, 18.105W)	A. J. Coello & M. Fernández-Mazuecos 30AC18	A, B	PP803758, PP803759, PP803760, PP803761	PP803919, PP803920, PP803921, PP803922
	Spain, Fuerteventura, El Cotillo (28.694N, 14.014W)	A. J. Coello & M. Fernández-Mazuecos 17AC19	B	PP803762, PP803763, PP803764, PP803765	PP803923, PP803924, PP803925, PP803926

Taxon	Locality (coordinates)	Voucher	Haplotypes	Accession numbers *psa*I-*aac*D	*psb*K-*trn*S
	Spain, Gran Canaria, Agaete (28.092N, 15.706W)	Y. Arjona 359YA15, 361YA15, 362YA15, 365YA15, 366YA15	D	PP803766, PP803767, PP803768, PP803769, PP803770	PP803927, PP803928, PP803929, PP803930, PP803931
	Spain, Gran Canaria, Cenobio de Valerón (28.143N, 15.607W)	A. J. Coello & M. Fernández-Mazuecos 50AC19	D	PP803771, PP803772, PP803773, PP803774, PP803775	PP803932, PP803933, PP803934, PP803935, PP803936
	Spain, Gran Canaria, Costa Ayala (28.134N, 15.485W)	A. J. Coello & M. Fernández-Mazuecos 45AC19	B	PP803776, PP803777, PP803778, PP803779, PP803780	PP803937, PP803938, PP803939, PP803940, PP803941
	Spain, Gran Canaria, Gáldar (28.159N, 15.703W)	Y. Arjona 355YA15, 356YA15, 357YA15, 358YA15	D	PP803781, PP803782, PP803783, PP803784	PP803942, PP803943, PP803944, PP803945
	Spain, Gran Canaria, Jinamar (28.038N, 15.407W)	Y. Arjona 613YA14, 615YA14, 616YA14, 617YA14	A	PP803785, PP803786, PP803787, PP803788	PP803946, PP803947, PP803948, PP803949
	Spain, Gran Canaria, Las Marciegas (27.997N, 15.815W)	A. J. Coello & M. Fernández-Mazuecos 58AC19	D	PP803789, PP803790, PP803791, PP803792, PP803793	PP803950, PP803951, PP803952, PP803953, PP803954

Taxon	Locality (coordinates)	Voucher	Haplotypes	**Accession numbers** *psa*I-*aac*D	*psb*K-*trn*S
	Spain, Gran Canaria, Playa de la Laja to Punta del Palo (28.057N, 15.418W)	A. J. Coello & M. Fernández-Mazuecos 34AC19	A	PP803794, PP803795, PP803796, PP803797, PP803798	PP803955, PP803956, PP803957, PP803958, PP803959
	Spain, Gran Canaria, Punta de los Cuervitos (27.878N, 15.388W)	A. J. Coello & M. Fernández-Mazuecos 36AC19	D	PP803799, PP803800, PP803801, PP803802, PP803803	PP803960, PP803961, PP803962, PP803963, PP803964
	Spain, Gran Canaria, Punta Sardina (28.164N, 15.708W)	A. J. Coello & M. Fernández-Mazuecos 53AC19	D	PP803804, PP803805, PP803806, PP803807, PP803808	PP803965, PP803966, PP803967, PP803968, PP803969
	Spain, Gran Canaria, Santa María de Guía (28.140N, 15.598W)	Y. Arjona 344YA15, 346YA15, 347YA15, 348YA15, 349YA15	D	PP803809, PP803810, PP803811, PP803812, PP803813	PP803970, PP803971, PP803972, PP803973, PP803974
	Spain, Gran Canaria, southern Agaete (28.089N, 15.703W)	A. J. Coello & M. Fernández-Mazuecos 54AC19	D	PP803814, PP803815, PP803816, PP803817, PP803818	PP803975, PP803976, PP803977, PP803978, PP803979

Taxon	Locality (coordinates)	Voucher	Haplotypes	Accession numbers *psa*I-*aac*D	*psb*K-*trn*S
	Spain, La Gomera, La Calera (28.100N, 17.347W)	A. J. Coello & M. Fernández-Mazuecos 10AC18	B, C	PP803819, PP803820, PP803821, PP803822, PP803823	PP803980, PP803981, PP803982, PP803983, PP803984
	Spain, La Gomera, Playa del Medio (28.038N, 17.181W)	M. Fernández-Mazuecos & A. J. Coello 14MF18	A, B	PP803824, PP803825, PP803826, PP803827	PP803985, PP803986, PP803987, PP803988
	Spain, La Gomera, Punta Llana (28.125N, 17.106W)	A. J. Coello & M. Fernández-Mazuecos 16AC18	C	PP803828, PP803829, PP803830, PP803831, PP803832	PP803989, PP803990, PP803991, PP803992, PP803993
	Spain, La Palma, Garafía (28.855N, 17.915W)	Y. Arjona 560YA14, 561YA14, 552YA14	A	PP803833, PP803834, PP803835	PP803994, PP803995, PP803996
	Spain, La Palma, Punta de Fuencaliente (28.455N, 17.843W)	A. J. Coello & M. Fernández-Mazuecos 79AC19	A	PP803836, PP803837, PP803838	PP803997, PP803998, PP803999
	Spain, La Palma, Punta Salinas (28.742N, 17.726W)	A. J. Coello & M. Fernández-Mazuecos 66AC19	A	PP803843, PP803844, PP803845, PP803846, PP803847	PP804004, PP804005, PP804006, PP804007, PP804008

Taxon	Locality (coordinates)	Voucher	Haplotypes	Accession numbers *psa*I-*aac*D	*psb*K-*trn*S
	Spain, La Palma, Puntallana (28.720N, 17.734W)	Y. Arjona 523YA14, 525YA14, 528YA14, 531YA14	A	PP803839, PP803840, PP803841, PP803842	PP804000, PP804001, PP804002, PP804003
	Spain, La Palma, Santa Cruz de La Palma (28.686N, 17.754W)	Y. Arjona 527YA14	A	PP803848	PP804009
	Spain, La Palma, Santo Domingo (28.831N, 17.944W)	A. J. Coello & M. Fernández-Mazuecos 75AC19	A	PP803849, PP803850, PP803851, PP803852, PP803853	PP804010, PP804011, PP804012, PP804013, PP804014
	Spain, La Palma, Tazacorte (28.650N, 17.945W)	A. J. Coello & M. Fernández-Mazuecos 84AC19	A	PP803854, PP803855, PP803856	PP804015, PP804016, PP804017
	Spain, La Palma, Bajada Puerto Naos (28.592N, 17.911W)	Y. Arjona 582YA14	A	PP803857	PP804018
	Spain, Lanzarote, Caleta de Famara (29.121N, 13.570W)	A. J. Coello & M. Fernández-Mazuecos 11AC19	B	PP803858, PP803859, PP803860, PP803861, PP803862	PP804019, PP804020, PP804021, PP804022, PP804023
	Spain, Lanzarote, Punta Prieta (29.119N, 13.641W)	A. J. Coello & M. Fernández-Mazuecos 12AC19	B	PP803863, PP803864, PP803865, PP803866	PP804024, PP804025, PP804026, PP804027

Taxon	Locality (coordinates)	Voucher	Haplotypes	Accession numbers	
				***psa*I-*aac*D**	***psb*K-*trn*S**
	Spain, Tenerife, Adeje (28.102N, 16.759W)	Y. Arjona 426YA14, 427YA14, 428YA14, 429YA14, 430YA14	C	PP803870, PP803871, PP803872, PP803873, PP803874	PP804031, PP804032, PP804033, PP804034, PP804035
	Spain, Tenerife, Buenavista (28.373N, 16.869W)	Y. Arjona 377YA14, 378YA14, 380YA14, 383YA14, 385YA14	A	PP803875, PP803876, PP803877, PP803878, PP803879	PP804036, PP804037, PP804038, PP804039, PP804040
	Spain, Tenerife, Güimar (28.300N, 16.369W)	A. J. Coello & M. Fernández-Mazuecos 25AC18	A, B	PP803885, PP803886, PP803887, PP803888, PP803889	PP804046, PP804047, PP804048, PP804049, PP804050
	Spain, Tenerife, El Tablado-Punta Prieta (28.263N, 16.390W)	Y. Arjona 475YA14, 477YA14, 479YA14, 481YA14, 482YA14	A	PP803880, PP803881, PP803882, PP803883, PP803884	PP804041, PP804042, PP804043, PP804044, PP804045
	Spain, Tenerife, La Caleta (28.102N, 16.759W)	M. Fernández-Mazuecos & A. J. Coello 7MF18	C	PP803890, PP803891, PP803892, PP803893, PP803894	PP804051, PP804052, PP804053, PP804054, PP804055

Taxon	Locality (coordinates)	Voucher	Haplotypes	Accession numbers *psa*I-*aac*D	*psb*K-*trn*S
	Spain, Tenerife, La Laguna (28.574N, 16.318W)	Y. Arjona 484YA14, 485YA14, 487YA14, 492YA14, 499YA14	A	PP803895, PP803896, PP803897, PP803898, PP803899	PP804056, PP804057, PP804058, PP804059, PP804060
	Spain, Tenerife, La Matanza (28.455N, 16.471W)	Y. Arjona 396YA14, 398YA14, 403YA14, 406YA14, 409YA14	A	PP803900, PP803901, PP803902, PP803903, PP803904	PP804061, PP804062, PP804063, PP804064, PP804065
	Spain, Tenerife, Red Mountain (28.031N, 16.547W)	A. J. Coello & M. Fernández-Mazuecos 24AC18	A	PP803905, PP803906	PP804066, PP804067
Molopospermum peloponnesiacum	Spain, Gerona, Ripollès (42.266N, 2.212E)	J. Calvo JC2743 (MA-01-00783092)	-	PP803907	PP804068
	Spain, Lérida, Vall d'Aran (42.808N, 0.806E)	A. Buira TB2688 (MA-01-00918164)	-	PP803908	PP804069

Table S2: Accession numbers of ITS sequences used in the phylogenetic analysis of *A. latifolia* and relatives (see main text). New sequences are indicated in bold.

Species	Accession number (ITS)
Annesorhiza filicaulis Eckl. & Zeyh.	DQ368832
Chamarea capensis (Thunb.) Eckl. & Zeyh.	DQ368837
Ezosciadium capense (Eckl. & Zeyh.) B.L.Burtt	AM982517
Itasina filifolia (Thunb.) Raf.	DQ368857
Astydamia latifolia (L.f.) Baill.	DQ368836, **PP794206**, **PP794207**, **PP794208**
Lichtensteinia crassijuga E.Mey. ex Sond.	EU434671
Molopospermum peloponnesiacum (L.) W.D.J.Koch	**PP794209**, **PP794210**
